# Integrated microbiome and metabolome analyses reveal spatial heterogeneity of medium-temperature Daqu and its potential impact on simulated strong-flavor baijiu fermentation

**DOI:** 10.1016/j.fochx.2026.104225

**Published:** 2026-07-20

**Authors:** Zhang Wen, Yu-Hua Wei, Hai-Yan Zhu, Liang Song, Da-Yong Han, Liang-Chen Guo, Chao-Jiu He, Zheng-Xiang Guo, Pei-Jie Han

**Affiliations:** a State Key Laboratory of Microbial Diversity and Innovative Utilization, Institute of Microbiology, Chinese Academy of Sciences, Beijing 100101, PR China; b College of Life Sciences, University of Chinese Academy of Sciences, Beijing 100049, PR China; c Jin Brand Nanxi Liquor Industry (Yibin) Co., Ltd., Yibin 629133, PR China

**Keywords:** Medium-temperature Daqu, Strong-flavor baijiu, Metabolic phenotype, Spatial heterogeneity, Functional heterogeneity, Solid-state simulated fermentation

## Abstract

Medium-temperature Daqu (MTD) exhibits pronounced spatial heterogeneity between its surface (QS) and inner (QI) portions, yet their functional differences remain poorly understood. This study compared physicochemical properties, metabolites, and species-level microbial communities of QS and QI and evaluated their impacts on simulated strong-flavor Baijiu (SFB) fermentation. QS exhibited higher saccharification and liquefaction activities and higher counts of lactic acid bacteria and yeasts, whereas QI was enriched in thermophilic molds and volatile compounds. Thirty-two microbial biomarkers differentiated QS and QI, and QS showed a more complex microbial co-occurrence pattern. Simulated fermentation revealed early bacterial divergence followed by convergence to *Acetilactobacillus jinshanensis.* During mid-to-late fermentation, QI showed higher levels of acids and esters than QS. Collectively, QS was mainly associated with substrate conversion and early fermentation initiation, whereas QI was more closely associated with flavor development during later fermentation stages. These findings provide valuable insights into MTD optimization and SFB quality control.

## Highlights

• MTD showed pronounced spatial heterogeneity in microbiota and metabolites.
• QS had higher saccharification and liquefaction activities than QI.
• QS favored early fermentation initiation during simulated SFB fermentation.
• QI promoted acid and ester accumulation during later fermentation.
• Spatial heterogeneity of MTD may affect Daqu function and SFB quality.

## 1 Introduction

Chinese Baijiu, one of the six major distilled spirits worldwide, is a traditional alcoholic beverage deeply embedded in Chinese dietary culture and social life (Ma et al., 2019). Based on flavor characteristics, Baijiu is classified into four basic types and eight derivative types (Chen et al., 2024). Among them, strong-flavor Baijiu (SFB) is the most widely consumed style, favored for its intense aroma, well-balanced flavor profile, and mellow, lingering mouthfeel, accounting for more than 70% of the total Baijiu market (Zheng & Han, 2016). Sichuan Province represents the core production region of SFB, where the brewing process follows a traditional solid-state fermentation paradigm. A mixture of sorghum, rice, glutinous rice, wheat, and corn is used as the raw material, with medium-temperature Daqu (MTD) serving as the starter, and anaerobic fermentation is carried out in mud pit (typically 6–8 m^3^ in volume) for 60–90 days, followed by distillation to obtain the final Baijiu product (Zheng & Han, 2016). Generally, MTD is produced in a naturally open environment, and its manufacturing process mainly includes raw material preparation (with wheat as the substrate), milling into particles, extrusion molding, fermentation, and storage (Ren et al., 2026). Consequently, the microbial communities in MTD are primarily derived from raw materials, production tools, and the surrounding environment. Under such conditions, MTD not only functions as the saccharification and fermentation starter, but also constitutes a highly diverse microbial community and part of the fermentation material; therefore, it is considered a key determinant of SFB quality and fermentation performance (Zou et al., 2018).

During fermentation, MTD develops a pronounced spatial heterogeneity, in which different regions within the Daqu brick experience distinct microenvironmental conditions, particularly with respect to temperature, oxygen availability, and moisture gradients (Tang et al., 2023). These environmental disparities lead to differential microbial growth dynamics and metabolite accumulation across spatial niches, ultimately giving rise to heterogeneous microbial community structures within a single Daqu brick. However, most previous studies have generally treated MTD as a homogeneous system. For example, Ren et al. (2026) characterized the properties of MTD of different grades by integrating microbiomic and metabolomic analyses; Zhang et al. (2025) investigated the heterogeneity of MTD across different geographical regions in China; Chen et al. (2026) examined the metabolic heterogeneity and biomarkers of MTD during the storage period from a temporal perspective; and Yang, Niu, et al. (2025) revealed the effects of soft wheat proportion on the microbial communities and quality attributes of MTD. Although these studies have expanded our understanding of MTD from multiple dimensions, they have largely overlooked the potential internal niche stratification within MTD, which may be a key driver of functional differentiation (Zhao et al., 2023). Indeed, although a few studies have reported differences in microbial communities between the surface and core regions of MTD during fermentation or storage (Jiang et al., 2025; Tang et al., 2023; Wen et al., 2024), this remains far from sufficient, as the specific functional roles of the surface and inner portions of MTD in SFB production are still unclear.

Therefore, this study combined physicochemical profiling, metabolomics, PacBio full-length sequencing, culturable microbial isolation, and laboratory-scale solid-state simulated fermentation system to investigate the differences in physicochemical properties, metabolites, and microbial communities across different parts of MTD and their potential impacts on SFB production. Specifically, orthogonal partial least squares discriminant analysis (OPLS-DA) was used to identify differential metabolites among MTD parts; co-occurrence network analysis and redundancy analysis (RDA) were applied to compare the internal and external factors shaping the microbial community structures of different MTD parts; Linear discriminant analysis effect size (LEfSe) analysis was employed to screen microbial biomarkers in each part; and principal coordinates analysis (PCoA) and OPLS-DA were used to examine differences in microbial community succession and volatile compounds during simulated fermentation using different MTD parts. These findings provide valuable insights for optimizing MTD production and improving quality control practices in the SFB industry.

## 2 Materials and methods

### 2.1 Sample collection

In April 2024, ten mature medium-temperature Daqu (MTD) bricks (approximately 30 cm × 20 cm × 7 cm in size), which had been stored for six months, were randomly selected from the same production batch at SFB distillery in Yibin, Sichuan Province, China. All MTD bricks were produced within the same production period following identical traditional processing procedures and stored under consistent conditions to ensure comparability. Based on previous observations that pronounced differences in physicochemical properties and microbial community structure exist between the surface layer (0–1 cm) and the inner portion of medium-temperature Daqu during fermentation (Wen et al., 2024), accordingly, the outermost 0–1 cm layer was defined as the surface portion (QS), whereas the remaining inner portion was defined as QI. It should be noted that QI represents the inner fraction of the Daqu brick and includes both intermediate and central regions rather than the strict geometric core. Additionally, a mixed sample (QM) was prepared by combining the surface and inner portions in their original proportion (Fig. S1). Each part was separately crushed, and approximately 500 g of Daqu powder was collected into sterile plastic bags, resulting in a total of 30 biological samples (10 QS, 10 QI, and 10 QM) for physicochemical characterization, microbial analysis, and representative sample preparation for the simulated fermentation experiment. For the simulated fermentation experiment, the ten biological replicates within each group (QS, QI, and QM) were thoroughly homogenized to generate one representative Daqu sample for each treatment. Each sample was divided into two portions. One portion was immediately processed for culturable microorganism counts, physicochemical measurements, and simulated fermentation experiments; the other was stored at −80 °C for genomic DNA extraction and metabolite analysis.

### 2.2 Physicochemical property analyses

Moisture, total acidity, starch, saccharifying activity (SA), liquefying activity (LA), fermentative activity (FA), and esterifying activity (EA) were determined in accordance with the Chinese industry standard QB/T 4257–2011. The pH value was measured using a digital pH meter (pH Meter AS700, Tokyo, Japan). Reducing sugar was detected by Fehling's titration method. The activities of neutral protease activity and acidic protease activity were measured using the Folin reagent method following the industry standard SB/T 10317–1999. All measurements were performed in triplicate to ensure data reliability and reproducibility.

### 2.3 Enumeration of culturable microorganisms

Culturable microorganisms in Daqu samples, including yeasts, mesophilic and thermophilic molds, lactic acid bacteria (LAB), *Bacillus*, and aerobic bacteria, were enumerated using the standard serial dilution and plating method. Yeasts were cultured on yeast extract peptone dextrose (YPD) agar supplemented with 2 mL/L acetic acid to suppress mold growth. Mesophilic molds were isolated on Rose Bengal agar, while thermophilic fungi were cultivated on potato dextrose agar (PDA) at 45 °C for 24–48 h. Other fungi were incubated at 30 °C for 48–72 h. To prevent bacterial growth, 0.2% chloramphenicol (100 mg/mL) was added to fungal media after autoclaving and cooling to approximately 60 °C. LAB were grown on Man, Rogosa, and Sharpe (MRS) agar. *Bacillus* were selectively isolated by preheating the sample suspension at 75 °C for 15 min before plating on nutrient agar. Aerobic bacteria were enumerated on R_2_A agar. For bacterial media, 0.1% amphotericin B (8 mg/mL) was added after autoclaving and cooling to approximately 60 °C to suppress fungal growth. All bacterial plates were incubated at 37 °C for 48–72 h. Colony counts were expressed as the logarithm of colony-forming units per gram, and all assays were performed in triplicate.

### 2.4 Solid-state simulated fermentation experiments

To investigate the potential functions of different MTD portions, representative QS, QI, and QM samples were prepared by thoroughly homogenizing the ten biological replicates within each group described in Section 2.1 and subsequently used for simulated fermentation. The grain mash was obtained from the same SFB distillery as the Daqu samples in Sichuan Province. For each sample, Daqu powder was added at 25% (*w*/w) following the traditional SFB brewing process and thoroughly mixed with the grain. Approximately 500 g of the mixture per replicate was transferred into sterile fermentation bags, vacuum-sealed, and incubated in the temperature-controlled incubator to simulate natural fermentation. The fermentation period lasted 60 days, and the temperature was adjusted every 24 h to mimic the ideal temperature-increasing profile of traditional SFB fermentation (Table S1). Each treatment (QS, QI, and QM) was fermented in triplicate at every sampling time point. Samples were collected at eight time points (0, 5, 10, 15, 20, 25, 35, and 60 d). At each sampling time, three biological replicates were obtained for each treatment (QS, QI, and QM), resulting in a total of 72 fermented grain samples (3 treatments × 8 time points × 3 biological replicates). In parallel, the blank control without Daqu addition was established and fermented under identical conditions to assess the baseline microbial community and metabolic profiles.

### 2.5 HPLC and HS-SPME-GC–MS analyses

The concentrations of sugars, alcohols, and organic acids in Daqu and fermented grain samples, as well as the contents of free amino acids in Daqu samples, were determined using high-performance liquid chromatography (HPLC). Sample pretreatment was performed following the protocol described by Wen et al. (2025). Sugars, alcohols, and organic acids were quantified using an HPLC system (LA-20 A, Shimadzu, Kyoto, Japan) equipped with the refractive index detector (RID-20 A), the photo diode array (PDA) detector, and an Aminex HPX-87H ion-exclusion column (300 mm × 7.8 mm, Bio-Rad, Contra Costa, USA). Chromatographic separation was achieved using 5 mmol/L sulfuric acid as the mobile phase at a flow rate of 0.6 mL/min, with the column temperature maintained at 60 °C and an injection volume of 10 μL. Free amino acids were analyzed on an HPLC system fitted with an AJS-02 amino acid analysis column (C_18_, 4.6 × 150 mm, 3 μm, Shimadzu) and the PDA detector, according to the method reported by Wen et al. (2025). All target compounds were identified and quantified based on their retention times and corresponding peak areas.

Volatile compounds (VOCs) were analyzed following sample pretreatment Wen et al. (2025) using headspace solid phase microextraction and gas chromatography–mass spectrometry (HS-SPME-GC–MS), according to the protocol described by Song et al. (2024). Retention indices (RIs) were obtained using an n-alkane standard mixture (C8-C20, Sigma-Aldrich). VOCs were putatively identified by matching mass spectra against the NIST 17 database, and only compounds with a matching degree >70% were retained and further verified by comparing their RIs with reference values. Semi-quantification of VOCs was performed based on normalized chromatographic peak areas relative to the internal standard (2-octanol), and the relative signal intensity of 2-octanol was used to calculate the percentage area of each peak.

### 2.6 Total DNA extraction, amplification and high throughput sequencing

Samples were first pretreated following the method described by Han et al. (2023), after which total genomic DNA was extracted according to the protocol reported by Luo et al. (2023). The quality of genomic DNA was assessed by agarose gel electrophoresis, and DNA concentrations were quantified using a Nano300 spectrophotometer and normalized to 50 ng/μL for all samples. The full-length bacterial 16S rRNA gene was amplified using the primer pair 27F (5′-AGA GTT TGA TCC TGG CTC AG-3′) and 1492R (5′-TAC GAC TTA ACC CCA ATC GC-3′), and the complete fungal internal transcribed spacer (ITS) region was amplified using the primer pair ITS1 (5′-TCC GTA GGT GAA CCT GCG G-3′) and ITS4 (5′-TCC TCC GCT TAT TGA TAT GC-3′). PCR amplification conditions and purification of amplicons were performed as previously described by Wei et al. (2025). Subsequently, sequencing was carried out on the PacBio Sequel II platform following the standard protocols provided by Annoroad Gene Technology Co., Ltd. (Beijing, China). Raw sequencing data were processed using CCS 6.0.0, VSEARCH 2.14, and USEARCH 11. After removal of barcodes and primers, chimeric sequences were identified against the UNITE CHIME reference database and discarded. The remaining sequences were de-replicated and denoised using the UNOISE3 algorithm to generate amplicon sequence variants (ASVs) at 100% sequence similarity with a minimum cluster size of 10. Taxonomic assignment was performed by comparison with the NCBI database using thresholds of >97% sequence identity and > 90% alignment coverage. Non-microbial sequences were removed before generating the final ASV table.

### 2.7 Processing of sequence data and statistical analysis

Bioinformatics analysis of the sequencing data was performed as previously described (Wen et al., 2024). All data analyses were performed in R software (version 4.2.2). The “vegan” package was used to evaluate α-diversity and β-diversity of samples, including Chao1 and Shannon indices, non-metric multidimensional scaling (NMDS), and the PCoA analysis based on Bray-Curtis distances. Co-occurrence networks were constructed based on Spearman correlation coefficients (|r| > 0.7, *P* < 0.01) and visualized in Gephi (version 0.9.2). The RDA was applied to examine the relationships between dominant bacterial and fungal species and physicochemical properties, and to evaluate their contributions to microbial community variation. Bubble plots and heatmaps were generated using the “ggpubr” and “pheatmap” packages. Differential metabolite analysis between groups was performed using orthogonal partial least squares discriminant analysis (OPLS-DA) with the “ropls” package in R. Model robustness and predictive performance were evaluated using 12-fold cross-validation, and potential overfitting was assessed by 200 permutation tests. Differential metabolites were screened based on the combined criteria of VIP scores >1, |log₂(Fold Change)| > 1, and adjusted *P* < 0.05, and were considered key metabolites distinguishing the groups (Wen et al., 2026). The LEfSe analysis was performed in an open-access pipeline (https://huttenhower.sph.harvard.edu/galaxy/) to identify differentially abundant taxa across groups, with thresholds set at LDA > 2.0 and P < 0.05 (Segata et al., 2011).

## 3 Results

### 3.1 Physicochemical properties and culturable microorganisms in different parts of MTD

**Fig. 1:**
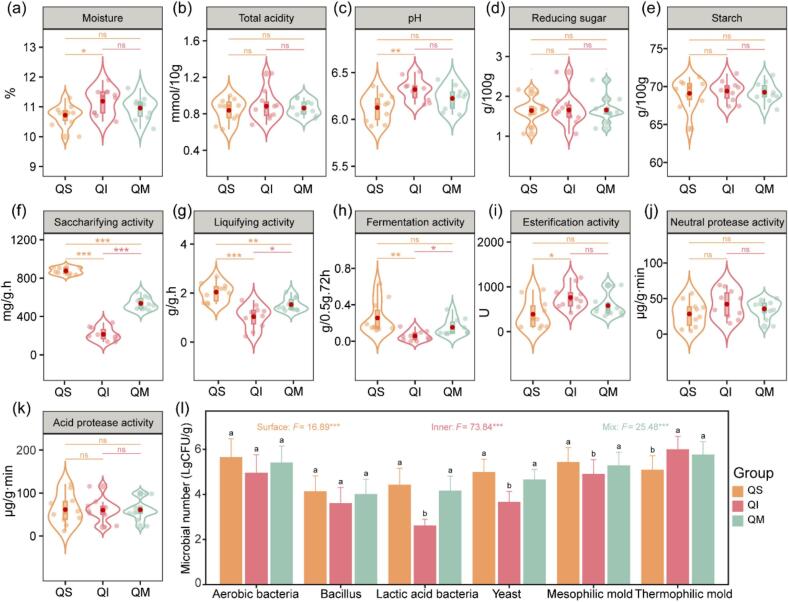
Differences in moisture (a), total acidity (b), pH (c), reducing sugar (d), starch (e), saccharifying activity (f), liquefying activity (g), fermentation activity (h), esterification activity (i), neutral protease activity (j), acid protease activity (k), and the abundance of six culturable microorganisms (l) among different parts of MTD. QS, QI, and QM represent the surface, inner, and mixed samples of MTD, respectively. NS, not significant; * *P* < 0.05; ** *P* < 0.01; *** *P* < 0.001.

The physicochemical properties and enzyme activities of MTD from different parts were evaluated, including moisture, total acidity, pH, reducing sugar, starch, saccharification, liquefaction, fermentation, esterification, neutral protease, and acid protease activities (Fig. 1). The results showed that the QI exhibited the highest moisture, pH, and esterification activity (Fig. 1a, c, i), whereas the QS displayed the highest saccharification, liquefaction, and fermentation activities (Fig. 1f-h). Notably, no significant differences (*P* > 0.05) were observed among the different parts (QS, QI, QM) with respect to total acidity, reducing sugar, starch, neutral protease, or acid protease activities (Fig. 1b, d, e, j, k).

Aerobic bacteria, *Bacillus*, LAB, yeast, mesophilic mold, and thermophilic mold were enumerated from different parts of MTD using six selective media. The results showed that the abundance of LAB, yeast, and mesophilic mold in the QI was significantly lower (*P* < 0.05) than in the QS and QM parts, whereas thermophilic mold exhibited the opposite trend. No significant differences (P > 0.05) were observed among the parts for aerobic bacteria and *Bacillus* (Fig. 1l). Furthermore, QI displayed the greatest variation among the six microbial groups (F = 73.84), indicating a pronounced imbalance in microbial composition within the QI.

### 3.2 Metabolite analysis among different parts of MTD

**Fig. 2:**
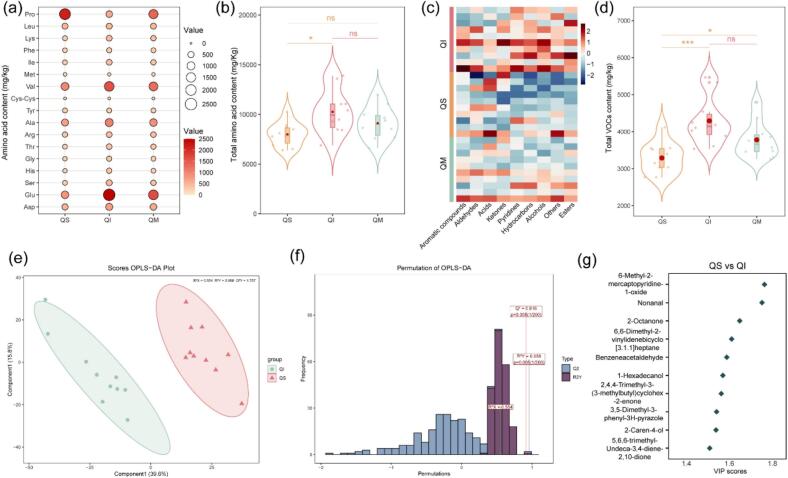
The contents of 17 free amino acids (a) and total free amino acids (b), as well as the semi-quantitative levels of nine categories of volatile flavor compounds (c) and total volatile flavor compounds (d) in QS, QI, and QM. OPLS-DA based on volatile flavor compound profiles of QS and QI, including the score plot (e) and permutation test results (f). Differential volatile compounds between QS and QI identified using a VIP threshold >1 (g).

The metabolite profiles of MTD from different parts were systematically characterized using HPLC and HS-SPME-GC–MS. Based on the analysis of 17 free amino acids, glutamic acid, proline, valine, alanine, and aspartic acid were identified as the dominant free amino acids across all Daqu parts. Among them, QI exhibited the highest total free amino acid content, which was significantly higher (*P* < 0.05) than that of QS, with glutamic acid contributing most prominently. In contrast, QS showed the lowest total free amino acid content but possessed a relatively higher level of proline (Fig. 2a-b). Further analysis of organic acids and sugars revealed that lactic acid and glycerol contents were highest in QS and were significantly greater (*P* < 0.05) than those in QI, whereas succinic acid displayed an opposite trend. Meanwhile, no significant differences (*P* > 0.05) were observed in citric acid or glucose contents among the different parts of MTD (Fig. S2).

Regarding volatile metabolites, a total of 118 compounds were identified by HS-SPME-GC–MS, including 7 pyridines, 2 acids, 13 aldehydes, 14 hydrocarbons, 25 esters, 14 ketones, 19 alcohols, 14 aromatic compounds, and 10 other compounds (Fig. S3). Overall, pronounced differentiation in volatile metabolite composition was observed among different MTD parts. Notably, the distribution patterns between QI and QS showed the most distinct differences: QI generally exhibited higher relative abundances of multiple compound classes, particularly pyridines, alcohols, and esters. In comparison, the volatile metabolite profile of QM was intermediate between QI and QS, displaying a clear transitional pattern (Fig. 2c). In addition, the total semi-quantitative level of volatile compounds in QI was significantly higher (P < 0.05) than that in QS (Fig. 2d). To further visualize these differences and to elucidate variations in volatile metabolites between QI and QS, OPLS-DA was applied for multivariate statistical analysis. As an effective supervised classification method widely used in metabolomics, OPLS-DA facilitates the interpretation of relationships between metabolite features and sample groupings (Ji et al., 2024; Kang et al., 2022). The OPLS-DA score plot demonstrated clear separation between QI and QS (Fig. 2e), indicating significant differences in their volatile compound compositions and reflecting pronounced spatial heterogeneity in potential metabolic functions. The OPLS-DA model exhibited good fitness and strong predictive ability (R^2^X = 0.554, R^2^Y = 0.958, and Q^2^ = 0.916), while a 200-permutation test confirmed its statistical significance and absence of overfitting (*P* = 0.005) (Fig. 2e-f). The top 10 discriminative volatile compounds between QI and QS were identified based on VIP > 1, |log₂ (Fold Change)| > 1, and adjusted *P* < 0.05. Among these, nonanal, and 2-octanone contributed most substantially to group discrimination and were therefore considered key compounds driving the observed differences in volatile metabolite profiles between the two Daqu parts (Fig. 2g).

### 3.3 Microbial community composition and diversity in different parts of MTD

**Fig. 3:**
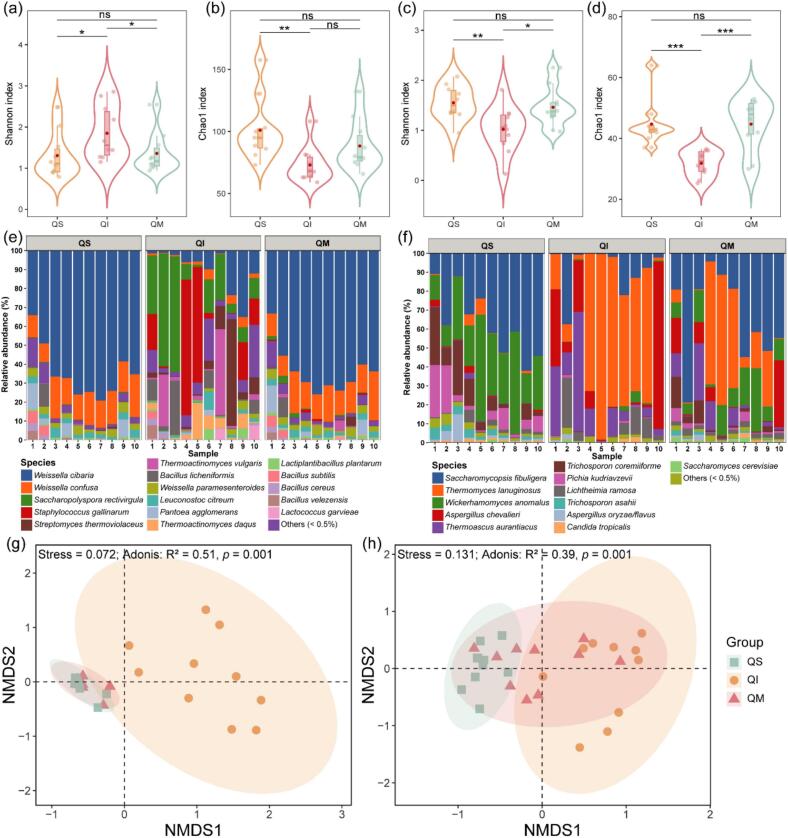
The α-diversity and community structure of microbial communities in different parts of MTD. The Shannon index (a) and Chao1 index (b) of bacterial communities, and the Shannon index (c) and Chao1 index (d) of fungal communities in QS, QI, and QM samples. Species-level bacterial (e) and fungal (f) community compositions across QS, QI, and QM, with species comprising less than 0.5% of relative abundance categorized as “Others”. The β-diversity of bacterial (g) and fungal (h) communities based on NMDS analysis.

After quality control, the numbers of effective bacterial sequences ranged from 45,216 to 66,642, with an average of 54,696, while the effective fungal sequences ranged from 51,436 to 63,845, with an average of 58,193. Rarefaction curves for all samples reached clear saturation plateaus, indicating that the sequencing depth was sufficient to capture the microbial community diversity and to support subsequent analyses (Fig. S4 a-b). The Shannon and Chao1 indices revealed differences in microbial diversity and richness among different parts of MTD. Specifically, the bacterial community in QI exhibited a significantly higher Shannon index but a lower Chao1 index than that in QS, indicating higher diversity but lower richness (Fig. 3a-b). In contrast, for fungi, both indices were significantly higher in QS than in QI. Overall, the microbial diversity and richness in QM were intermediate between QS and QI (Fig. 3c-d). At the species level, the bacterial communities in QS and QM were dominated by *Weissella cibaria* and *Weissella confusa*, whereas the bacterial community in QI was more complex, with *Saccharopolyspora rectivirgula*, *Staphylococcus gallinarum*, *Streptomyces thermoviolaceus*, and *Thermoactinomyces vulgaris* as the predominant species (Fig. 3e).

Correspondingly, the fungal community in QS was primarily composed of *Saccharomycopsis fibuligera*, *Wickerhamomyces anomalus*, *Trichosporon coremiiforme*, and *Pichia kudriavzevii*. In contrast, *Thermomyces lanuginosus*, *S. fibuligera*, and *Aspergillus chevalieri* predominated in the fungal communities of QI and QM (Fig. 3f). Furthermore, NMDS based on Bray-Curtis distances was employed to evaluate differences in microbial community composition among the different parts of MTD. The results showed that the bacterial communities of QS and QM clustered closely, whereas those of QI were clearly separated from the other two (Fig. 3g). For fungal communities, a distinct separation was observed between QS and QI, while QM exhibited partial overlap with both groups (Fig. 3h).

The LEfSe analysis was further applied to identify microbial biomarkers differentiating MTD from different parts (LDA > 2.0 and *P* < 0.05). Overall, the largest number of differential biomarkers was detected between QI and QS (Fig. S5c, f). In contrast, fewer discriminative taxa were identified between QM and the other two groups, suggesting that QM exhibits a transitional microbial composition (Fig. S5a-b, d-e). Specifically, in the bacterial community, QS was significantly enriched in LAB taxa, particularly *W. cibaria* and *W. confusa*. By comparison, QI was characterized by the enrichment of multiple thermotolerant or thermophilic bacterial taxa, including *T. daqus* and *T. vulgaris*, reflecting the adaptation of the QI bacterial community to higher-temperature conditions (Fig. S5c). In terms of the fungal community, QS was mainly characterized by yeast taxa such as *W. anomalus*, *S. fibuligera*, and *P. kudriavzevii*. In contrast, QI showed significant enrichment of thermophilic fungi, including *T. lanuginosus* and *Thermoascus aurantiacus*, highlighting pronounced differences in fungal ecological niches among different parts of MTD (Fig. S5f).

### 3.4 Microbial co-occurrence patterns and physicochemical factors associated with microbial communities in different parts of MTD

**Fig. 4:**
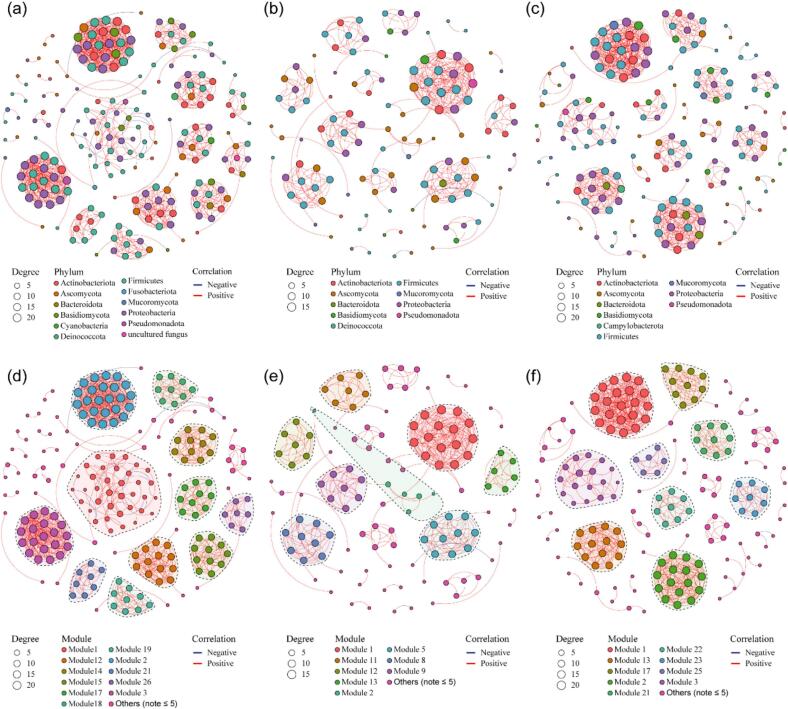
Species-level microbial co-occurrence networks of QS (a), QI (b), and QM (c), and their corresponding module-based networks (d-f) (|r| > 0.7, P < 0.01). In panels (a-c), each node represents a microbial species, and node colors indicate taxonomic affiliation at the phylum level.

To further characterize microbial co-occurrence patterns and network complexity in different parts of MTD, microbial co-occurrence networks were constructed, revealing that the co-occurrence networks in QS, QI, and QM were all dominated by positive correlations (Fig. 4a-c). Among them, the QS network was the largest, comprising 212 nodes and 920 edges, with 98.59% of the associations being positive, while 13 negative correlations were still detected. In contrast, the QI network was the smallest, containing 124 nodes and 345 edges, and exhibited only two negative correlations, resulting in the highest proportion of positive associations (99.42%). The QM network showed intermediate complexity, with 178 nodes and 659 edges, and the positive correlation proportion of 99.24% (Table S2). In terms of network connectivity, QS displayed the higher average degree (8.68) and larger network diameter (8.11) than QI (5.56 and 2.75) and QM (7.40 and 3.65), suggesting more complex co-occurrence patterns and denser network connectivity among microbial taxa (Table S2). By contrast, QI exhibited the shortest average path length (1.03), suggesting a more compact network topology. Module-based network partitioning further highlighted structural differences among the three sample types (Fig. 4d-f). All networks showed pronounced modular organization, with modularity indices exceeding 0.83. QS exhibited slightly higher modularity value (0.842), accompanied by relatively elevated centralization betweenness (0.008), suggesting the presence of taxa with relatively higher topological importance within the network (Table S2). In comparison, QI and QM showed lower centralization values, indicating more evenly distributed associations across nodes. Collectively, the higher node number, edge number, and average degree observed in QS indicate more complex microbial co-occurrence patterns, whereas QI and QM exhibited smaller networks with predominantly positive co-occurrence patterns.

**Fig. 5:**
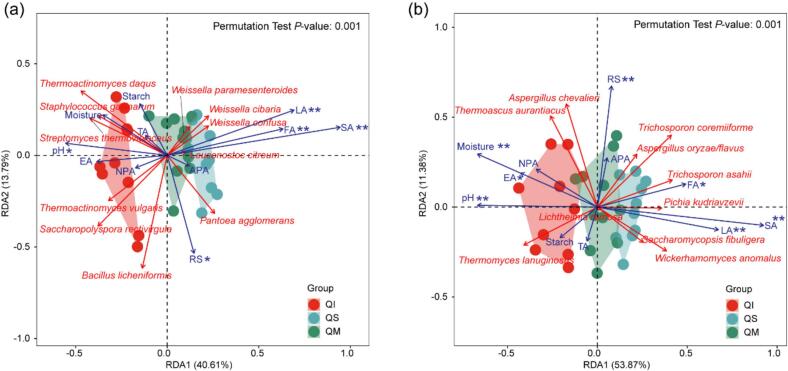
Redundancy analysis (RDA) showing the impacts of physicochemical properties on the microbial community compositions of QS, QI, and QM in different parts of MTD. *, 0.01 < P < 0.05; **, 0.001 < *P* < 0.01.

RDA was employed to analyze the associations among 11 dominant bacterial species, 10 dominant fungal species, and 11 key physicochemical factors, thereby identifying physicochemical factors associated with microbial community variation in different parts of MTD. For the bacterial community, three *Weissella* species, *Leuconostoc citreum*, and *Pantoea agglomerans* were identified as the bacterial taxa most strongly associated with QS. These taxa exhibited significant positive correlations with LA, FA, and SA, suggesting that the QS bacterial community is closely associated with starch conversion and the initiation of fermentation processes. In contrast, *T. daqus*, *S. gallinarum*, and *S. thermoviolaceus* were the bacterial taxa most strongly associated with the QI community, and all showed significant positive correlations with pH (Fig. 5a). For the fungal community, five yeast species represented by *Trichosporon asahii* were the fungal taxa most strongly associated with QS and were significantly positively correlated with LA, FA, and SA, highlighting a close association between yeast populations and enzyme systems involved in starch degradation and fermentation. By comparison, the fungal community in QI was predominantly associated with the thermophilic molds *T. lanuginosus* and *T. aurantiacus*. These fungi showed significant positive correlations with moisture, pH, and EA, which may reflect their adaptation to high-moisture environments and their potential association with enzymatic reactions and material transformation during MTD fermentation (Fig. 5b). Overall, the RDA results demonstrate pronounced differentiation in microbial composition and the physicochemical factors associated with microbial community variation among different parts of MTD.

### 3.5 Microbial community composition and diversity during simulated fermentation of different parts of MTD

**Fig. 6:**
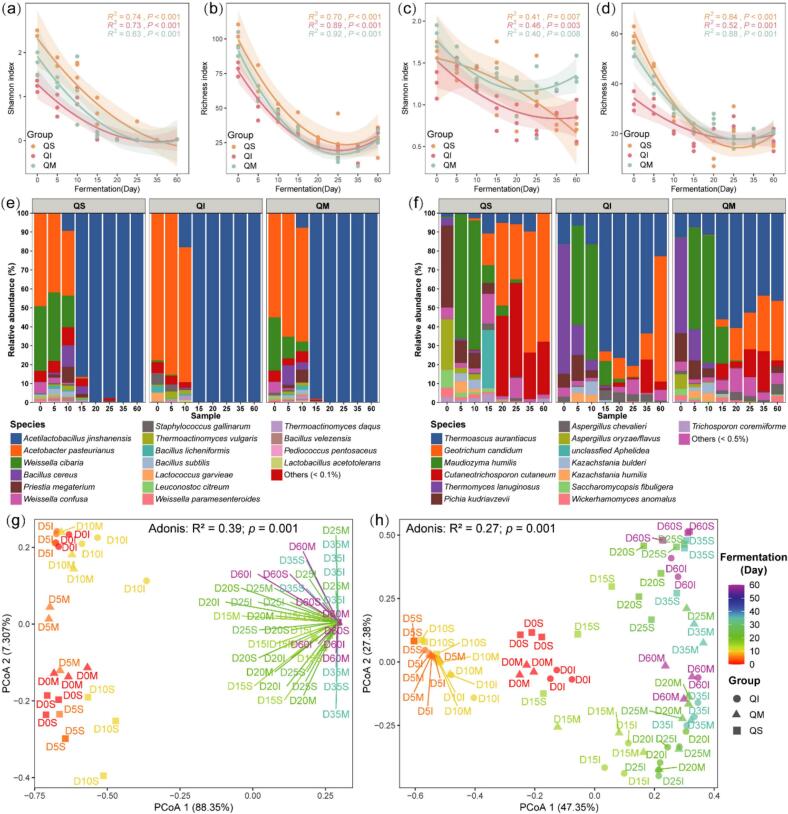
Diversity and structure of the microbial community during the simulated fermentation of QS, QI, and QM. The α-diversity indices of bacterial communities at the species level (a-b) and fungal communities at the species level (c-d). Dynamic changes of bacterial (e) and fungal (f) communities at the species level, with species comprising less than 0.1% or 0.5% relative abundance categorized as “Others”. The PCoA analysis of bacterial (g) and fungal (h) communities based on the Bray-Curtis dissimilarity matrix at the species level.

Building on the above results showing pronounced differences in metabolic characteristics and microbial composition among QS, QI, and QM, laboratory-scale solid-state simulated fermentations were conducted to further evaluate their effects on the fermentation process of SFB. The fermentation process was dynamically monitored by sequential sampling (Fig. 6). The α-diversity analysis demonstrated that different parts of MTD distinctly influenced microbial diversity during fermentation. For bacterial communities, QS exhibited the highest Shannon and Richness indices during days 0–30 of fermentation, followed by QM, whereas QI consistently showed the lowest diversity levels (Fig. 6a-b). In contrast, for fungal communities, QI displayed the lowest Shannon and Richness indices at most fermentation stages, while QS and QM showed more pronounced temporal fluctuations (Fig. 6c-d). Species-level community composition analysis further revealed distinct microbial succession trajectories among treatments (Fig. 6e-f). Notably, in the blank control without MTD addition at day 0, the bacterial community was predominantly composed of *Acetilactobacillus jinshanensis* (Fig. S6a), whereas the fungal community was mainly dominated by *P. kudriavzevii* (Fig. S6b). By the end of fermentation (60 d), the microbial community in the blank control was mainly composed of *A. jinshanensis*, *Schizosaccharomyces pombe*, and *Maudiozyma humilis* (Fig. S6a-b), providing a baseline reference for evaluating the effects of MTD inoculation. During the early fermentation stage (0–10 d), the bacterial communities of QS and QM were mainly dominated by *Acetobacter pasteurianus* and *W. cibaria*, whereas QI was primarily dominated by *A. pasteurianus*. As fermentation progressed to 15–60 d, bacterial communities in all three treatments gradually converged and became dominated by a limited number of taxa, particularly *A. jinshanensis* (Fig. 6e). Regarding fungal communities, QI and QM were predominantly occupied by *T. lanuginosus* and *M. humilis* during 0–10 d, and subsequently shifted to dominance by *T. aurantiacus* and *Geotrichum candidum* during 15–60 d. In contrast, QS was characterized by *P. kudriavzevii* and *M. humilis* at the early stage, followed by *G. candidum* and *Cutaneotrichosporon cutaneum* at later stages (Fig. 6f). The β-diversity patterns were consistent with these observations. The PCoA analysis showed that bacterial communities of QS at 0–10 d clustered closely with QM at 0 d, while QI at 0–10 d was more similar to QM at 10 d; by 15–60 d, bacterial community structures among QS, QI, and QM became increasingly similar (Fig. 6g). For fungal communities, QS, QI, and QM clustered closely during days 5–10 of fermentation, whereas during days 15–60, QM and QI exhibited higher similarity, with QS clearly separated (Fig. 6h). Overall, these results indicate that different parts of MTD are associated with significant differences in microbial diversity, community composition, and successional patterns during simulated SFB fermentation, highlighting potential functional heterogeneity within the fermentation system.

### 3.6 Metabolite analysis during simulated fermentation of different parts of MTD

**Fig. 7:**
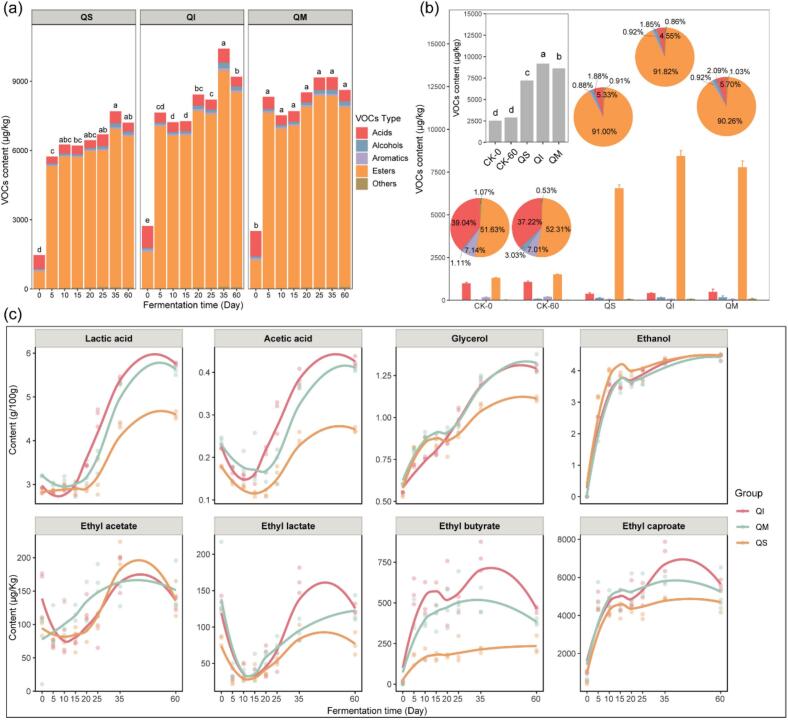
Changes in flavor-related metabolites during simulated strong-flavor Baijiu fermentation using different parts of MTD. (a) Dynamic changes in five categories of volatile flavor compounds. (b) Semi-quantitative levels of the five categories of volatile flavor compounds and total volatile flavor compounds in the blank control at the beginning and end of fermentation, and in QS, QI, and QM at the end of fermentation. (c) Dynamic changes in major organic acids and alcohols (lactic acid, acetic acid, glycerol, and ethanol) and key esters (ethyl acetate, ethyl lactate, ethyl butyrate, and ethyl hexanoate).

Consistent with the observed differences in microbial communities, different parts of MTD markedly influenced the composition and succession of metabolites during simulated fermentation. A total of 84 flavor compounds were detected throughout the fermentation process, including 44 esters, 12 alcohols, 11 acids, 6 aromatic compounds, 3 aldehydes, 3 hydrocarbons, and 5 other compounds (Fig. S7). Among these, esters were persistently dominant, followed by acids and alcohols (Fig. 7a). In the blank control without Daqu addition, the flavor profiles at the initial and final stages of fermentation remained relatively stable, being mainly composed of esters (51.63% and 52.31%, respectively) and acids (39.04% and 37.22%, respectively) (Fig. 7b). In contrast, simulated fermentations supplemented with different parts of MTD exhibited pronounced esterification characteristics at the end of fermentation, with esters accounting for 91.00%, 91.82%, and 90.26% in QS, QI, and QM, respectively. Further comparison of the total semi-quantitative levels of volatile flavor compounds showed that QI exhibited the highest level at the end of fermentation, followed by QM and QS. All three treatments showed significantly higher levels than the control group (CK-60), with significant differences among treatments (Fig. 7b). At the overall metabolic level, OPLS-DA further confirmed the pronounced differences in volatile metabolite profiles between QS and QI. The score plot showed a clear separation between the two groups (Fig. S8a), indicating that different parts of MTD substantially reshaped the volatile metabolic features during simulated fermentation. The OPLS-DA model exhibited good fitness and satisfactory predictive ability (R^2^X = 0.896, R^2^Y = 0.650, and Q^2^ = 0.795). Furthermore, the 200-permutation test confirmed the statistical significance of the model and indicated no evident overfitting (*P* = 0.005), demonstrating the robustness and reliability of the analysis (Fig. S8b). Based on these results, the dynamic changes of major metabolites were further compared to elucidate differences in key flavor compound formation (Fig. 7c). Except for ethyl acetate, which accumulated to relatively higher levels in QS during the late fermentation stage, ethyl lactate, ethyl butyrate, and ethyl hexanoate were consistently higher in QI than in QS throughout most of the fermentation process. In addition, during the mid-to-late fermentation stages (20–60 d), lactic acid, acetic acid, and glycerol accumulated to markedly higher levels in QI than in QS, whereas ethanol content was slightly higher in QS than in QI during the mid-fermentation stage (5–25 d). Overall, different parts of MTD significantly modulated the accumulation patterns of key metabolites during fermentation, with particularly pronounced differences in ester formation.

## 4 Discussion

The findings of this study support a spatial functional differentiation framework for medium-temperature Daqu (MTD), in which the surface portion (QS) appears to be primarily associated with substrate conversion and early-stage fermentation initiation, whereas the inner portion (QI) is more closely associated with organic acid accumulation and ester formation during later fermentation stages. This observed functional differentiation may be associated with distinct physicochemical microenvironments and microbial assemblages between the surface and inner regions of MTD.

The present study demonstrates pronounced spatial heterogeneity within MTD, reflected in physicochemical properties, microbial composition, metabolite profiles, and fermentation performance. Unlike previous studies that primarily focused on describing the internal heterogeneity of MTD during fermentation or storage, this study further elucidates the effects of surface and inner portions of MTD on microbial succession and metabolite profiles during SFB fermentation through simulated fermentation experiments. The observed functional differentiation between QS and QI was consistently supported by differences in physicochemical properties, microbial composition, and fermentation performance, suggesting that spatial heterogeneity within MTD may influence both substrate conversion and flavor formation. In addition, QI also showed higher levels of major organic acids, particularly lactic acid. Collectively, these findings provide new insights into the spatial ecological organization of microbial communities within MTD and suggest that internal niche differentiation may contribute to variation in the functional potential of Daqu during SFB fermentation.

The enzyme activities in Daqu originate from the microorganisms enriched within it and largely determine the quality of Daqu (Huang et al., 2024; Zhu et al., 2024). Consistent with previous studies (Hou et al., 2022; Jiang et al., 2025; Wen et al., 2024), the present study demonstrated that the saccharification and liquefaction activities of QS were significantly higher than those of QI (Fig. 1f-g). Functionally, LA reflects the ability of microorganisms to degrade starch into intermediate products such as dextrins, whereas saccharification activity represents their capacity to further convert these intermediates into reducing sugars, particularly glucose (Huang et al., 2024). Combined with microbial community analysis, *W. cibaria*, *W. confusa*, and *S. fibuligera*, which were significantly enriched in QS (Fig. 3e-f), showed strong positive correlations with both saccharification and liquefaction activities (Fig. 5). Previous studies have demonstrated that *W. cibaria* and *W. confusa* not only participate in starch degradation but also play important roles in glucose metabolism (Yang et al., 2021). Meanwhile, *S. fibuligera* is capable of efficiently secreting α-amylase and β-glucosidase and is recognized as a key saccharifying microorganism in cereal-based fermentation systems (Wang et al., 2021). Therefore, the enrichment of these functional taxa in QS may partially explain its enhanced capacity for starch degradation and sugar production from a microbial functional perspective. Collectively, the elevated saccharification and liquefaction activities in QS suggest that the surface portion may provide a stronger capacity for starch degradation and fermentable substrate release, thereby facilitating early fermentation initiation. Furthermore, the enrichment of lactic acid bacteria in QS was associated with higher lactic acid levels (Fig. 1l, S2), which may be linked to lower pH values (Fig. 1c), whereas the enrichment of yeast taxa may be associated with higher fermentation activity (Fig. 1h, l). Among them, *S. fibuligera* can produce aroma compounds, esters, and ethanol during fermentation, thereby improving liquor quality (Xie et al., 2021). In addition, *P. kudriavzevii* is noted for its desirable aroma- and enzyme-producing characteristics (Li et al., 2023), and is regarded as a key functional yeast in Baijiu production and has been identified as a major contributor to liquor-producing and fermenting power in low-temperature Daqu (Zhang et al., 2024). The co-enrichment of these yeast taxa in QS may partly explain its relatively stronger fermentation performance.

In contrast to QS, QI exhibited a significantly higher moisture content (Fig. 1a). This can be primarily attributed to limited water evaporation in the inner region, as the surface layer is more exposed to air and undergoes continuous moisture loss. In addition, temperature gradients during fermentation may promote moisture migration and accumulation in the inner portion. The elevated moisture content in QI may increase water availability for microbial metabolism, thereby potentially enhancing enzymatic reactions, which in turn promotes enzymatic reactions. Such conditions favor the enrichment of thermophilic fungi (Fig. 1l), including *T. lanuginosus*, *T. aurantiacus* and *A. chevalieri* which are recognized as important contributors to esterification activity in Daqu (Tie et al., 2025; Yang, Tie, et al., 2025). Consistently, QI exhibited higher EA (Fig. 1i) and accumulated greater amounts of key flavor compounds, particularly ethyl esters (Fig. 2c-d). These findings suggest that the moisture-enriched microenvironment in QI may contribute to differences in microbial composition and ester accumulation during fermentation.

Biotic and abiotic factors may jointly contribute to the spatial differentiation of microbial communities within MTD, thereby contributing to the stable formation of Daqu enzymes and flavor profiles (Ma et al., 2022; Zhu et al., 2022). From the perspective of biotic interactions, the co-occurrence networks of different MTD parts all exhibited high modularity (modularity >0.83), indicating pronounced modular organization within the co-occurrence networks (Table S2). Such modular co-occurrence patterns suggest structured association patterns among microbial taxa rather than random distribution (Hernandez et al., 2021; Yuan et al., 2021). Notably, the QS network displayed higher complexity and stronger connectivity (Fig. 4, Table S2), suggesting more complex co-occurrence patterns among microbial taxa. Previous studies have shown that microbial interactions can influence microbial fitness, population dynamics, and functional potential (Berg et al., 2020). Therefore, differences in microbial co-occurrence patterns between QS and QI may be associated with the observed differences in enzymatic activities and metabolic characteristics. From the perspective of abiotic factors, physicochemical properties were strongly associated with the functional differentiation of microbial communities and may reflect potential environmental filtering effects. The QS microbiota exhibited a stronger substrate conversion potential and is primarily involved in starch degradation and the initiation of fermentation (Fig. 5). In contrast, the QI community was more closely associated with high-temperature, high-moisture, and relatively enclosed conditions, which may contribute to increased flavor compound transformation and accumulation during fermentation (Fig. 5). This environmentally driven functional divergence reflects distinct resource utilization strategies and metabolic partitioning among microbial communities occupying different spatial niches. Together, these findings suggest that microbial associations and environmental heterogeneity jointly shape niche partitioning within MTD.

Although distinct dominant bacterial communities were observed among different simulated fermentation systems at the early stage of fermentation, the community structures gradually converged as fermentation progressed, ultimately becoming dominated by *A. jinshanensis* (Fig. 6e). This successional pattern is consistent with previous observations in SFB fermentation systems and reflects a pronounced environmental filtering effect under progressively acidified conditions (Lin et al., 2025; Xu et al., 2025). As a key lactic acid-producing bacterium, *A. jinshanensis* exhibits strong acidophilic and acid-tolerant characteristics. These traits may allow it to outcompete other microorganisms during the later fermentation stages and promote continued organic acid accumulation (Chen et al., 2025). In contrast, *A. pasteurianus* preferentially proliferated in the QI system during the early fermentation stage, which likely accelerated the initial production of acetic acid. The continuous accumulation of lactic and acetic acids subsequently exerts dual effects on the fermentation system: lowering pH to suppress competing microorganisms and providing essential precursors for the biosynthesis of esters and medium-chain fatty acids (Deng et al., 2020; Wu et al., 2019). In this context, the QI system exhibited higher levels of organic acid accumulation during the mid-to-late stages of fermentation, thereby providing a sufficient substrate pool for ester synthesis. Meanwhile, QI showed the highest esterification enzyme activity (Fig. 1i), which may further enhance the conversion efficiency of organic acids into ester compounds. In addition, *T. aurantiacus*, which became dominant in the later fermentation stage (Fig. 3f), not only possesses the ability to secrete cellulases and xylanases, but also exhibits potential for amylase production and participation in ester formation (Tang et al., 2024). This suggests that it may simultaneously contribute to substrate degradation and flavor formation during SFB fermentation. Overall, the QI system displayed more pronounced organic acid accumulation and esterification capacity throughout fermentation, which may account for the relatively higher levels of ethyl lactate, ethyl butyrate, and ethyl hexanoate observed in the later stage (Fig. 7c).

The pronounced differentiation observed among different parts of MTD in this study provides a basis for understanding their potential roles in SFB fermentation. From a practical perspective, the selective use or proportional blending of different MTD parts may help achieve a better balance between saccharification capacity and flavor formation, thereby potentially improving fermentation stability and promoting the production of key flavor compounds. On this basis, the optimal proportion of QS and QI may be further adjusted based on fermentation performance. Furthermore, these findings suggest that optimization of the physical structure of MTD may influence the relative contributions of different functional regions. For example, increasing the surface area and reducing the thickness of MTD brick may enhance QS-associated functions, while decreasing the surface area and increasing brick thickness may favor QI-associated functions. However, these scenarios remain hypothetical and require validation in pilot-scale and industrial fermentation before being translated into production practice. Overall, the spatial heterogeneity identified in this study provides a useful framework for future optimization of MTD production and application, and offers directions for subsequent process refinement.

Notwithstanding the potential practical implications of this study, several limitations should be carefully considered. First, all samples were collected from a single production batch at one distillery. Consequently, the observed physicochemical, microbial, and metabolic characteristics may partly reflect batch-specific production conditions, which may limit the generalizability of the findings. Second, methodological limitations should also be acknowledged. Although full-length 16S rRNA gene and ITS sequencing improved taxonomic resolution, species-level assignments should still be interpreted cautiously, particularly for closely related taxa. Moreover, amplicon sequencing primarily reflects microbial community composition rather than direct metabolic activity or functional expression. Therefore, the functional roles inferred for specific taxa require further validation through cultivation-based approaches or multi-omics analyses. Furthermore, the present sampling strategy divided MTD into surface and inner portions, and thus the QI fraction may include both intermediate and central regions rather than representing the true geometric center of the Daqu block. Given the likely existence of continuous physicochemical and microbial gradients from the surface toward the interior, future studies using finer spatial sampling are warranted to further resolve internal heterogeneity. Meanwhile, QM was prepared by mixing QS and QI according to their original mass proportions, which may result in a stronger influence of the QI fraction on its overall characteristics. Furthermore, statistical approaches used in this study, including RDA, co-occurrence network analysis, LEfSe, and OPLS-DA, primarily reveal association patterns rather than direct causal relationships. Functional roles and microbial interactions inferred from these analyses require further validation through cultivation-based experiments, functional assays, or controlled perturbation studies. Finally, the simulated fermentation system used in this study was designed as a controlled laboratory-scale model and does not fully replicate the complex conditions of industrial strong-flavor Baijiu fermentation, such as pit mud-associated microbiota, large-scale spatial gradients, and dynamic heat and gas transfer. Therefore, although this study provides a useful framework for understanding the functional heterogeneity of MTD, the practical applicability of these findings requires further validation under industrial fermentation conditions.

## 5 Conclusions

This study systematically revealed pronounced differences in physicochemical properties, metabolites, and microbial composition between QI and QS of MTD. By integrating simulated fermentation, the study provides further evidence that QI and QS contribute differently to microbial succession and flavor formation in SFB fermentation. Collectively, QS appears to contribute primarily to substrate conversion and early-stage fermentation initiation, whereas QI contributes more to acid accumulation and ester formation during later fermentation stages. The microbial community compositions of QS and QI were markedly distinct, and thirty-two differential microbial biomarkers were identified by LEfSe analysis. The microbial co-occurrence network of QS exhibited greater complexity than that of QI. Simulated fermentation demonstrated that the dominant bacteria differed between QS and QI during the early stage (days 0–10), whereas *A. jinshanensis* predominated in both during the middle and late stages (days 15–60). Meanwhile, during days 20–60, *T. aurantiacus* dominated the fungal communities in QI and QM, whereas QS was mainly dominated by *G. candidum* and *C. cutaneum*. Metabolite analysis revealed that QI was the major contributor to lactic acid, acetic acid, and key esters (ethyl lactate, ethyl hexanoate, and ethyl butyrate) during the late fermentation stage (days 25–60). The preferential accumulation of organic acids and esters in QI suggests that the inner portion may contribute more strongly to flavor development during the middle and late stages of fermentation, particularly through pathways associated with acid production and esterification. These findings provide a basis for understanding the functional heterogeneity of MTD and may inform future strategies for Daqu utilization and quality control. However, validation under industrial fermentation conditions is required before practical implementation.

## CRediT authorship contribution statement

**Zhang Wen:** Writing – review & editing, Writing – original draft, Visualization, Investigation, Data curation, Conceptualization. **Yu-Hua Wei:** Writing – original draft, Methodology, Investigation, Data curation. **Hai-Yan Zhu:** Methodology, Data curation. **Liang Song:** Methodology, Data curation. **Da-Yong Han:** Methodology, Data curation. **Liang-Chen Guo:** Methodology, Data curation. **Chao-Jiu He:** Validation, Supervision, Conceptualization. **Zheng-Xiang Guo:** Investigation. **Pei-Jie Han:** Writing – review & editing, Project administration, Funding acquisition, Conceptualization.

## Declaration of competing interest

The authors declare that they have no known competing financial interests or personal relationships that could have appeared to influence the work reported in this paper.

## Data Availability

Data will be made available on request.
