# Supplementary material for: Integrated microbiome and metabolome analyses reveal spatial heterogeneity of medium-temperature Daqu and its potential impact on simulated strong-flavor baijiu fermentation
Source: Food Chem X. 2026 Jul 20;38:104225. doi: 10.1016/j.fochx.2026.104225 (PMC13396755; doi:10.1016/j.fochx.2026.104225)
Supplement: Supplementary file 1 — Supplementary material 1 [file mmc1.docx]

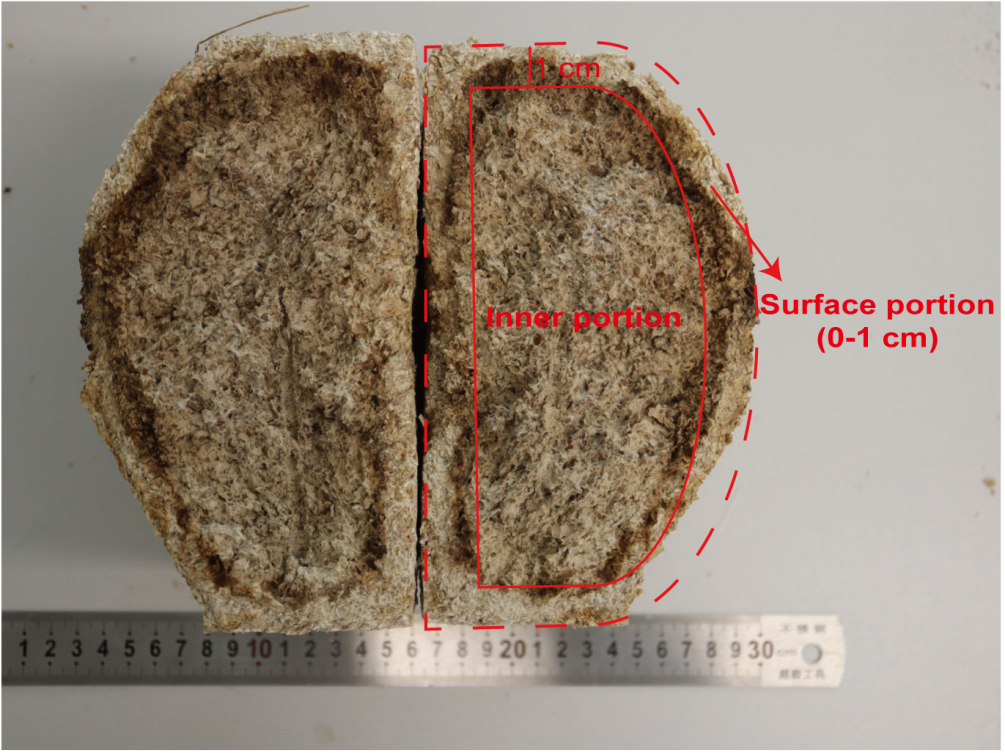


**Figure S1.** A schematic diagram illustrating the different parts of MTD.


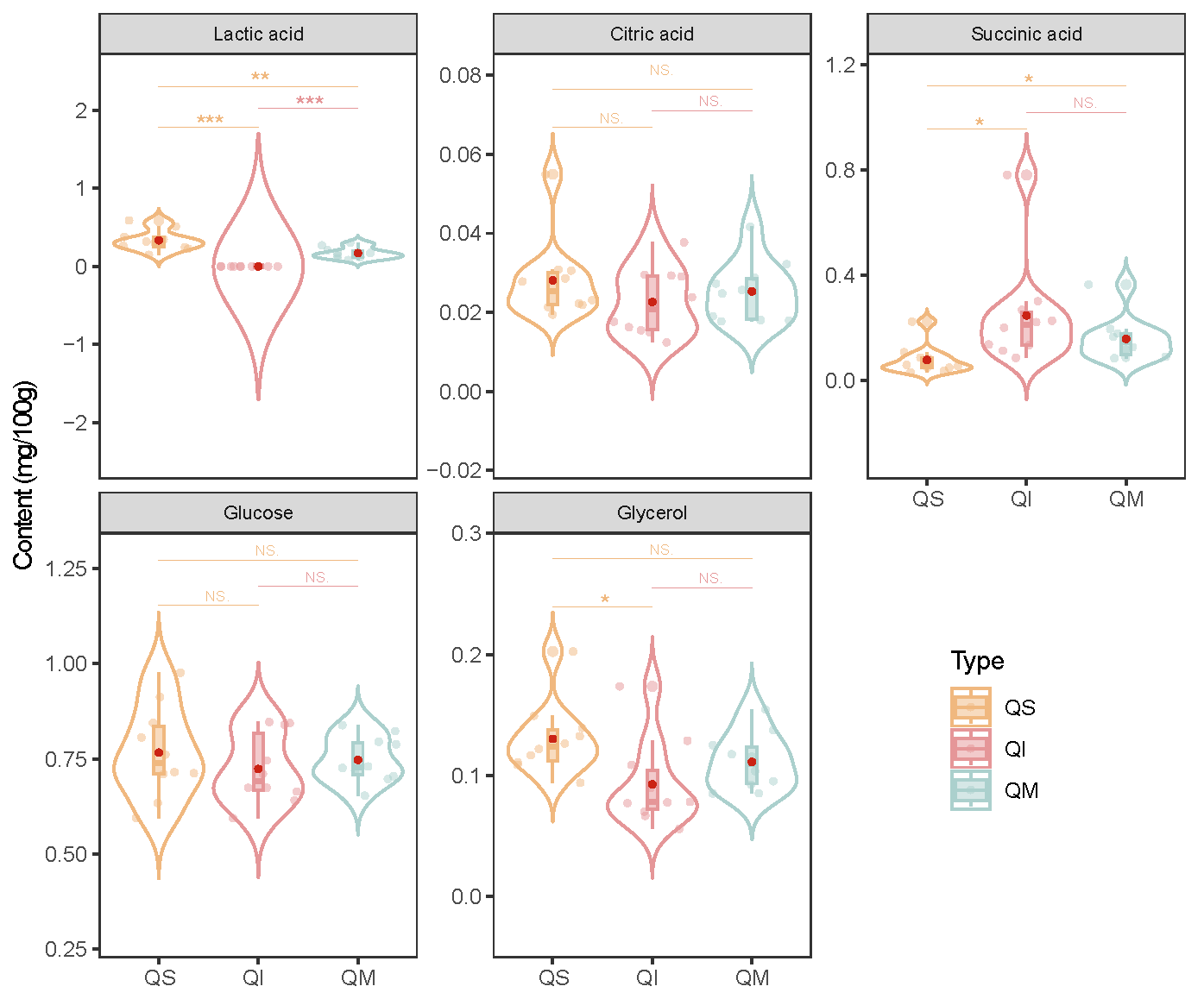


**Figure S2.** Differences in lactic acid, citric acid, succinic acid, glucose, and glycerol among different parts of MTD. NS, not significant; * P < 0.05; ** P < 0.01; *** P < 0.001.


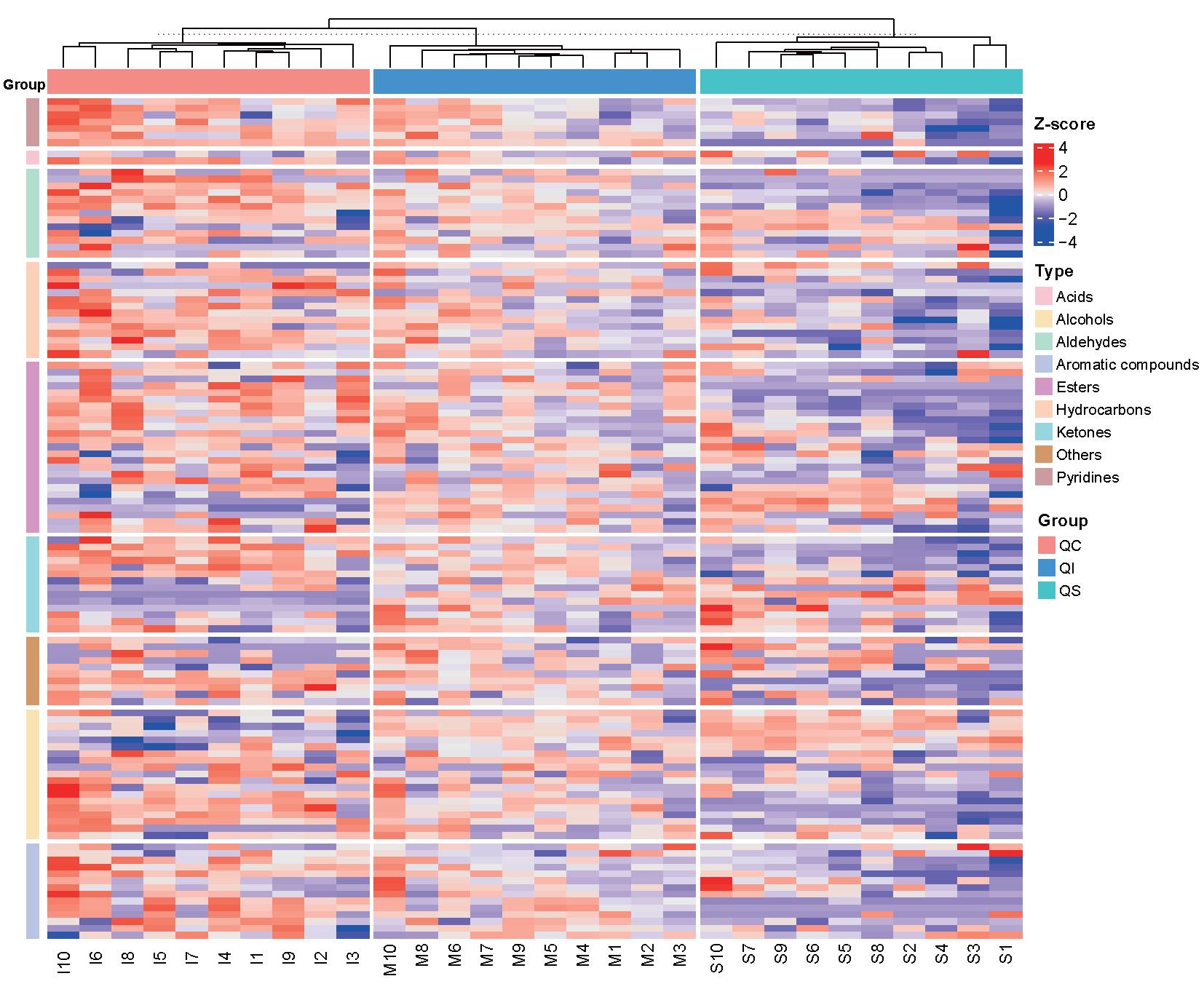


**Figure S3.** Semi-quantitative analysis of nine categories of volatile flavor compounds in different parts of MTD.


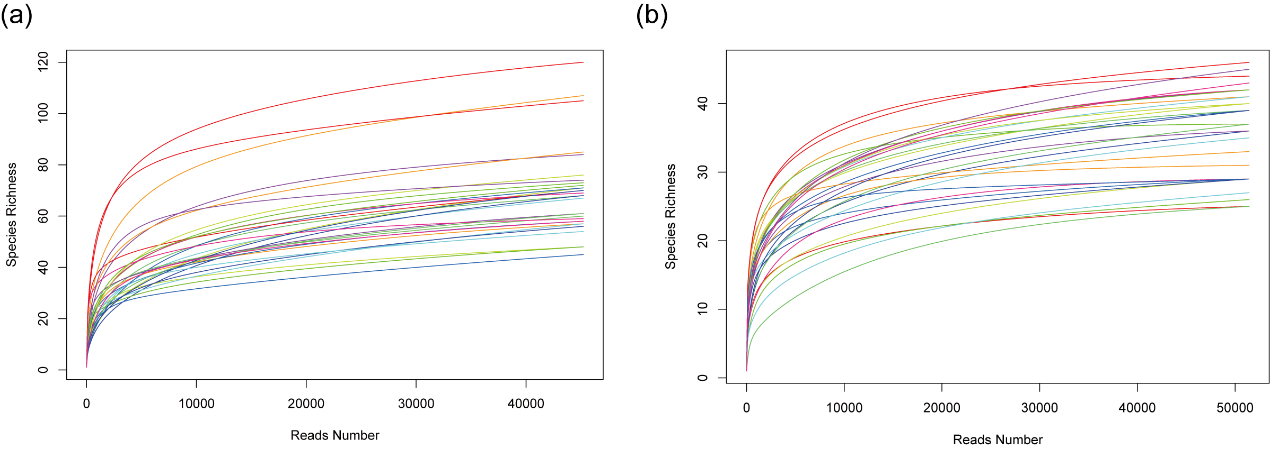


**Figure S4.** Rarefaction curves for bacterial (a) and fungal (b) communities in different parts of MTD.


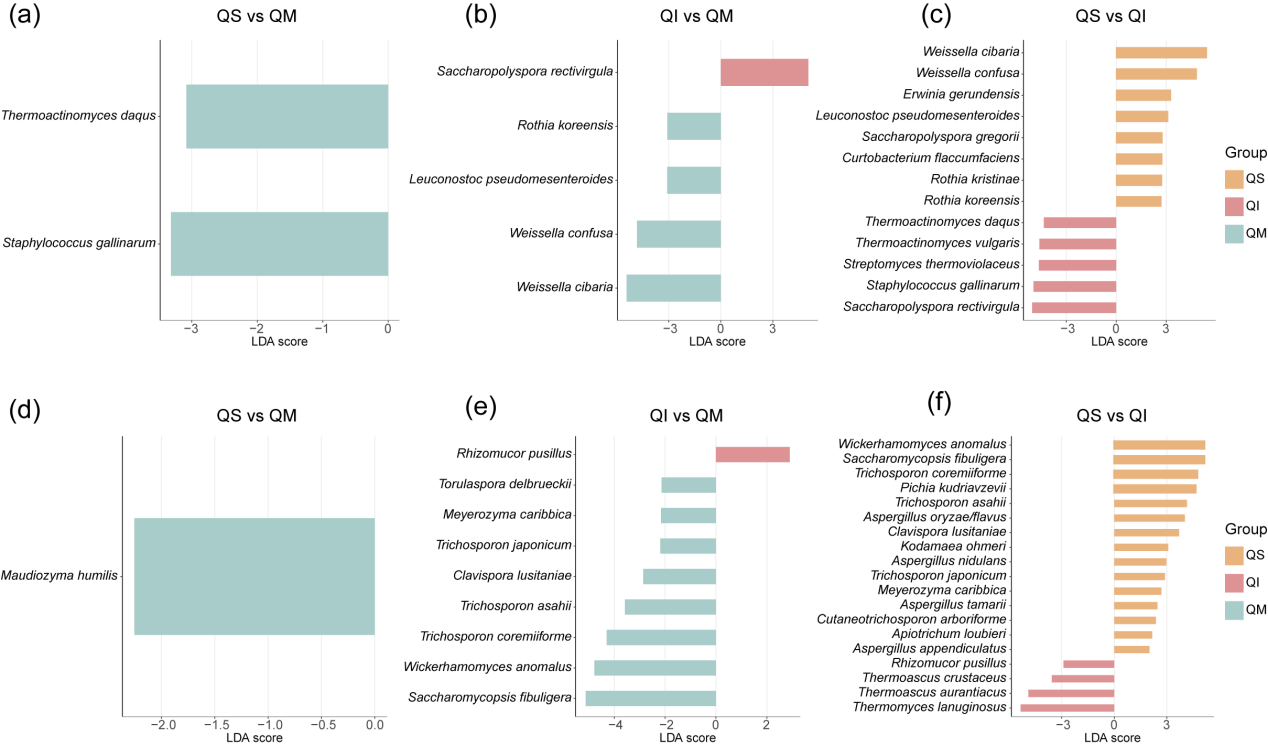


**Figure S5.** Differential bacterial species (a-c) and fungal species (d-f) between pairwise comparisons of QS, QI, and QM identified by LEfSe analysis (LDA > 2, P < 0.05). The LDA (linear discriminant analysis) scores reflect the degree of discrimination for each species.


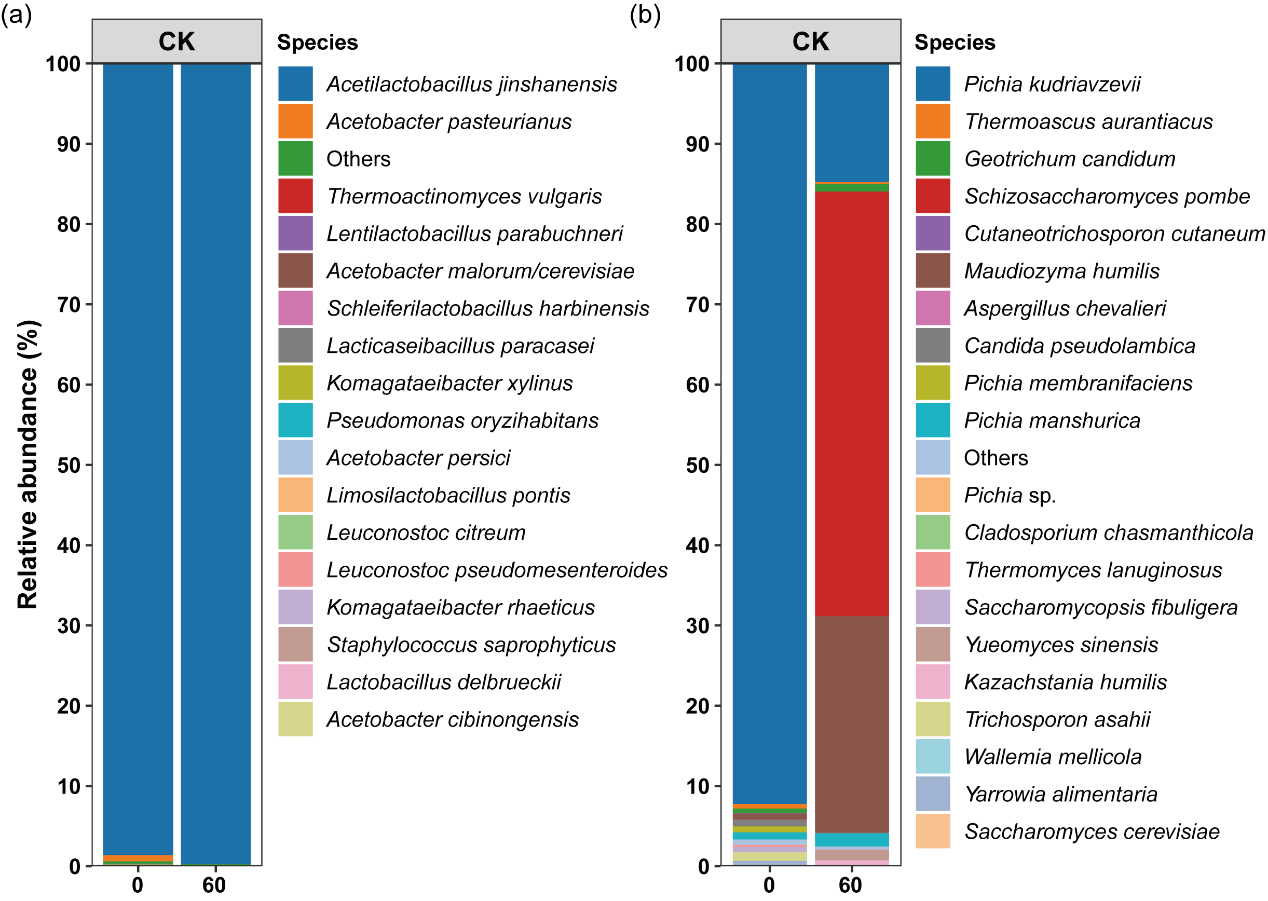


**Figure S6.** Species-level bacterial (a) and fungal (b) community compositions of the blank control without MTD at the start (day 0) and end (day 60) of the simulated fermentation.


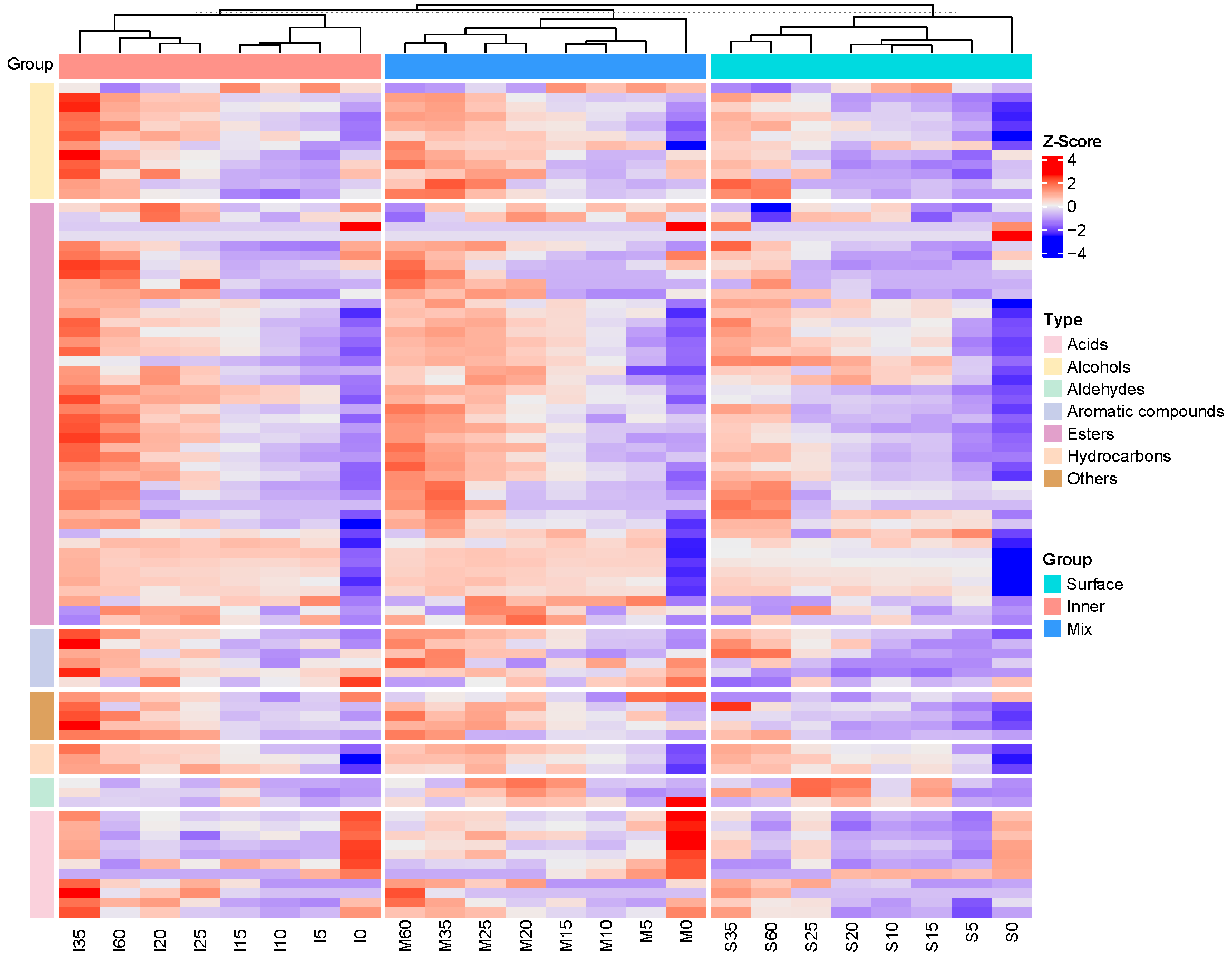


**Figure S7.** Temporal changes in semi-quantitative contents of 84 volatile flavor compounds (seven categories) during simulated fermentation of strong-flavor Baijiu using different parts of MTD.


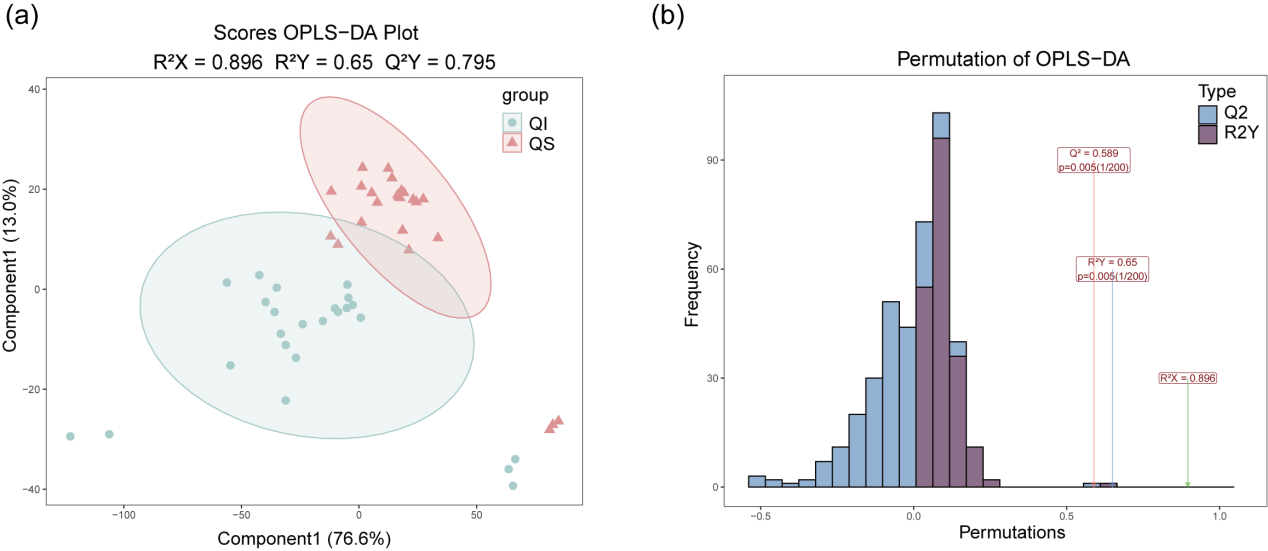


**Figure S8.** The OPLS-DA model constructed based on the volatile flavor compound profiles during the simulated fermentation of strong-flavor Baijiu using QS and QI, including the OPLS-DA score plot (a) and permutation test results (b).
