# Supplementary material for: Integrated microbiome and metabolome analyses reveal spatial heterogeneity of medium-temperature Daqu and its potential impact on simulated strong-flavor baijiu fermentation
Source: Food Chem X. 2026 Jul 20;38:104225. doi: 10.1016/j.fochx.2026.104225 (PMC13396755; doi:10.1016/j.fochx.2026.104225)
Supplement: Supplementary file 2 — Supplementary material 2 [file mmc2.docx]

**Table S1** Temperature control conditions for simulated strong-flavor Baijiu fermentation based on the actual fermentation heating curve

| Date | Fermentation time (days) | Temperature (℃) |
| --- | --- | --- |
| 23 Apr,2024 | 0 | 16 |
| 24 Apr,2024 | 1 | 17 |
| 25 Apr,2024 | 2 | 18 |
| 26 Apr,2024 | 3 | 19 |
| 27 Apr,2024 | 4 | 19.5 |
| 28 Apr,2024 | 5 | 20 |
| 29 Apr,2024 | 6 | 23 |
| 30 Apr,2024 | 7 | 26 |
| 1 May,2024 | 8 | 27 |
| 2 May,2024 | 9 | 29 |
| 3 May,2024 | 10 | 29.5 |
| 4 May,2024 | 11 | 30 |
| 5 May,2024 | 12 | 30 |
| 6 May,2024 | 13 | 30.5 |
| 7 May,2024 | 14 | 30.5 |
| 8 May,2024 | 15 | 30.5 |
| 9 May,2024 | 16 | 30 |
| 10 May,2024 | 17 | 30 |
| 11 May,2024 | 18 | 29.5 |
| 12 May,2024 | 19 | 29.5 |
| 13 May,2024 | 20 | 29 |
| 14 May,2024 | 21 | 29 |
| 15 May,2024 | 22 | 29 |
| 16 May,2024 | 23 | 28.5 |
| 17 May,2024 | 24 | 28.5 |
| 18 May,2024 | 25 | 28 |
| 19 May,2024 | 26 | 28 |
| 20 May,2024 | 27 | 28 |
| 21 May,2024 | 28 | 27.5 |
| 22 May,2024 | 29 | 27.5 |
| 23 May,2024 | 30 | 27 |
| 24 May,2024 | 31 | 27.00 |
| 25 May,2024 | 32 | 27.00 |
| 26 May,2024 | 33 | 27.00 |
| 27 May,2024 | 34 | 27 |
| 28 May,2024 | 35 | 27 |
| 29 May,2024 | 36 | 27 |
| 30 May,2024 | 37 | 27 |
| 31 May,2024 | 38 | 26.5 |
| 1 June,2024 | 39 | 26.5 |
| 2 June,2024 | 40 | 26.5 |
| 3 June,2024 | 41 | 26.5 |
| 4 June,2024 | 42 | 26.5 |
| 5 June,2024 | 43 | 26.5 |
| 6 June,2024 | 44 | 26.5 |
| 7 June,2024 | 45 | 26.5 |
| 8 June,2024 | 46 | 26 |
| 9 June,2024 | 47 | 26 |
| 10 June,2024 | 48 | 26 |
| 11 June,2024 | 49 | 26 |
| 12 June,2024 | 50 | 26 |
| 13 June,2024 | 51 | 26.5 |
| 14 June,2024 | 52 | 26.5 |
| 15 June,2024 | 53 | 26.5 |
| 16 June,2024 | 54 | 26.5 |
| 17 June,2024 | 55 | 26.5 |
| 18 June,2024 | 56 | 25 |
| 19 June,2024 | 57 | 25 |
| 20 June,2024 | 58 | 25 |
| 21 June,2024 | 59 | 25 |
| 22 June,2024 | 60 | 25 |

**Table S2** The topological parameters of microbial co-occurrence networks in different parts of MTD (QS, QI, and QM)

|  | QS | QI | QM |
| --- | --- | --- | --- |
| Nodes | 212 | 124 | 178 |
| Edges | 920 | 345 | 659 |
| Positive correlation | 907 | 343 | 654 |
| Negative correlation | 13 | 2 | 5 |
| Positive correlation percentage | 98.58695652 | 99.42028986 | 99.24127466 |
| Negative correlation percentage | 1.413043478 | 0.579710145 | 0.758725341 |
| Average degree | 8.679245283 | 5.564516129 | 7.404494382 |
| Average path length | 1.927283687 | 1.031337816 | 1.162327098 |
| Network diameter | 8.109125219 | 2.75420876 | 3.652389004 |
| Network density | 0.041133864 | 0.045239969 | 0.041833302 |
| Clustering coefficient | 0.989404285 | 0.997523877 | 0.989996152 |
| Centralization betweenness | 0.00795494 | 0.000924292 | 0.002728279 |
| Degree centralization | 0.067870875 | 0.076711251 | 0.071161049 |
| Modularity | 0.841677694 | 0.832379752 | 0.835620255 |
